# Gill regeneration in the mayfly *Cloeon* uncovers new molecular pathways in insect regeneration

**DOI:** 10.1098/rsob.240118

**Published:** 2024-11-27

**Authors:** Carlos A. Martin-Blanco, Pablo Navarro, José Esteban-Collado, Florenci Serras, Isabel Almudi, Fernando Casares

**Affiliations:** ^1^ CABD (Andalusian Center for Developmental Biology), CSIC/Universidad Pablo de Olavide/Junta de Andalucía, Seville 41013, Spain; ^2^ Department of Genetics, Microbiology and Statistics, Universitat de Barcelona, Diagonal 643, 08028, Barcelona, Spain; ^3^ Institute of Biomedicine of the University of Barcelona (IBUB), Diagonal 643, 08028, Barcelona, Spain; ^4^ Institut de Recerca de la Biodiversitat (IRBio), Universitat de Barcelona, Diagonal, 643, 08028, Barcelona, Spain

**Keywords:** insect regeneration, gills, *Cloeon dipterum*, *Drosophila*, neddylation, growth control

## Abstract

The capacity to regenerate lost organs is widespread among animals, and yet the number of species in which regeneration has been experimentally probed using molecular and functional assays is very small. This is also the case for insects, for which we still lack a complete picture of their regeneration mechanisms and the extent of their conservation. Here, we contribute to filling this gap by investigating regeneration in the mayfly *Cloeon dipterum*. We focus on the abdominal gills of *Cloeon* nymphs, which are critical for osmoregulation and gas exchange. After amputation, gills re-grow faster than they do during normal development. Direct cell count and EdU assays indicate that growth acceleration involves an uniform increase in cell proliferation throughout the gill, rather than a localized growth zone. Accordingly, transcriptomic analysis reveals an early enrichment in cell cycle-related genes. Other gene classes are also enriched in regenerating gills, including protein neddylation and other proteostatic processes. We then showed the conservation of these mechanisms by functionally testing protein neddylation, the activin signalling pathway or the mRNA-binding protein Lin28, among other genes, in *Drosophila* larval/pupal wing regeneration. Globally, our results contribute to elucidating regeneration mechanisms in mayflies and the conservation of mechanisms involved in regeneration across insects.

## 1. Introduction

Regeneration allows restoring function, partially or fully, after an organ has been lost or damaged. This capacity is widespread, although scattered, along the animal tree of life, with some groups (or even species within groups) being able to fully regenerate their organs, while others are capable only of partial regeneration, or none at all [1–3].

Among animals, crustacea and insects (Pancrustacea) are excellent regenerators, especially of their limbs [4,5]. Specifically within insects, regeneration has been described in species belonging to 38 genera [6]. Insects secrete an exoskeleton, so that growth is allowed by the regular shedding, or moulting, of the exoskeleton until the animal reaches adulthood. Furthermore, as a general rule, regeneration in insects requires moulting. Thus, as adult pterygote insects do not moult, regeneration only occurs during the larval or nymphal period.

Despite the broad distribution of regenerative capacity within insects, the search for molecular and cellular mechanisms involved in insect appendage regeneration has been restricted to a small set of species (reviewed in [4,7]). This research has identified a number of genetic components and pathways that seem to be shared during limb regeneration in insects such as crickets and cockroaches or flies [8–14], and includes the early involvement of the Jun stress response and the JAK/STAT pathways, the Hippo and insulin/IGF growth-control pathways, the activation of patterning signalling pathways, such as wg/Wnt, hh/Shh, EGF, dpp/BMP, Notch and Toll, the usage of transcription factors known to be critical in proximo-distal leg patterning, such as *Dll*, *dac* or *hth* and the requirement of the planar *Ds/ft* polarity pathway [7]. However, there are major gaps in our understanding of insect regeneration and the extent to which regenerative mechanisms are conserved across species and organs. Several studies have found that genes and pathways previously known to play a major role during limb development are also involved in limb regeneration, raising the possibility that regeneration recapitulates the developmental programme [15]. However, the discovery of damage/regeneration-specific regulatory elements suggests that the gene networks responsible for the early phases of wound healing and regeneration differ from development [16–18]. Moreover, recent work on the malacostracan species *Parhyale hawaiensis* (both insects and malacostracans belong to the clade Pancrustacea) shows that, although leg development and regeneration share a similar set of expressed genes, the temporal deployment of these genes differs [19].

**Figure 1.**
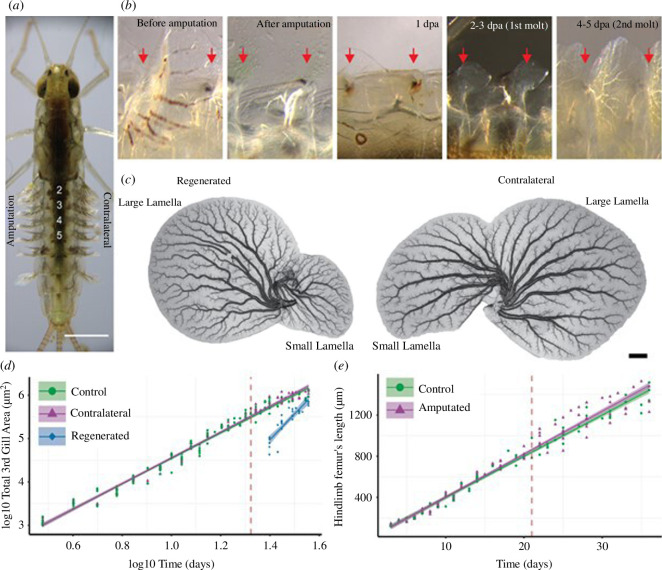
Accelerated growth of regenerating gills. (*a*) 21 days post-eclosion female nymph before amputation. Numbers in white mark abdominal segments that were selected for gill amputation. Gills on the left flank were amputated by pulling while the contralateral ones on the right flank remained unoperated. Scale bar: 1 mm. (*b*) Dorsal view of third segment gill regeneration after total amputation. Anterior to the left and distal to the top. From left to right: segment before and after amputation, 1 day post-amputation (1 dpa) shows a melanization clot formed in the abdomen where the gill was connected to the body. In addition, the trachea that connected the gill to the tracheal system has disintegrated. The fourth panel shows a regenerated gill that appears after moulting once post-amputation, approximately 2 dpa. After a second moult**,** approximately 4–5 dpa, regenerating gills already have a visible tracheal tree. Red arrows indicate the point where the gills (third and fourth) connect to the abdomen. (*c*) Whole mount gills from a last nymphal instar. Scale bar: 100 µm. Note the gill’s large and small lamella. (*d*) Linear models of control (green), contralateral (purple) and regenerated (blue) gills representing log10 area versus log10 time (*R*^2^: 0.9893, *p*‐value = 2.2 × 10^−16^. Regenerated gill estimate: 1.86424 *p*‐value = 1.55 × 10^−9^). (*e*) Linear model of the hindlimb femur mean length (between left and right for each individual) over time (*R*^2^ = 0.9735, *p*‐value = 2.2 × 10^−16^; no significant differences between groups).

The pond olive mayfly *Cloeon dipterum* is a freshwater insect that only emerges from the water as a reproductive, flying adult [20]. The aquatic juveniles, or nymphs, carry a pair of gills on each of their first seven abdominal segments (A1−7). These gills are flat, paddle-like organs joined to the abdomen through a thin hinge (figure 1*a*). The gills on A1−6 have two lamellae each and are motile, while the gills on A7 are mono-lamellated and non-motile [21]. Gills are tracheated and thought to be involved in gas exchange/respiration (e.g. [22–24]; but see [25]; figure 1*a−c*), in osmoregulation through specialized chloride cells [26,27], and in chemosensing [28]. Gill regeneration in *Cloeon* nymphs was reported more than a century ago [29]. However, *Cloeon’s* regenerative capacity extends to other appendages, including legs, antennas, wing rudiments and terminal cerci [20].

There are two particular aspects of gills we found of special interest. One is that gills suffer frequent autotomy (*sensu* Maginnis [6]): in our cultures, we observe that gills often detach from the body and remain trapped in the shed cuticle during moulting [20]. This amputation happens at the base of the gill. After such amputation, a normal-looking gill regenerates within 5–9 days (figure 1*b*,*c*). Given the functional importance of gills for respiration, osmoregulation and chemical sensing, this regenerative capacity is likely to be important for survival. Another interesting aspect is the relation between gills and wings: the abdominal gills of mayflies have been suggested to be serially homologous to wings [30–33] (reviewed in [26]), a relationship that has been recently supported by transcriptomic analyses [28].

In this article, we shed light on these questions by describing the dynamics of gill regeneration in terms of morphology, cell and transcriptional dynamics in *Cloeon dipterum* nymphs. Our study extends the evolutionary range of insects where regeneration has been probed using molecular approaches, spanning approximately 400 million years of insect evolution [34,35]. Our transcriptomic analysis identifies processes, pathways and genes already known to be involved in the regeneration of appendages in other insects, but also molecules and processes not previously associated with insect limb regeneration. We tested the functional role of orthologues of some of these genes, including components of the proteasome, the extracellular matrix and the activin pathway, in *Drosophila* larval/pupal wing regeneration. These experiments confirm the involvement of these candidates in regeneration and identify new regeneration-related mechanisms. The conservation of regeneration mechanisms between flies and mayflies supports the idea that such mechanisms may be widely conserved, at least within insects.

## 2. Results

### 2.1. Accelerated growth of regenerating gills

Previous studies in the ladybug *Coccinella septempunctata* showed that these insects are able to regenerate their legs, but with a developmental cost that results in larger individuals with developmental delays [36]. By contrast, the stick insect *Sipyloidea sipylus* reduces the size of its wings upon leg regeneration [37]. Thus, our first experiment asked how the dynamics of regeneration compared to normal gill development, and whether gill regeneration had an impact on organismal growth. To address these questions, we separated sibling nymphs into two batches. The first was a control group raised in standard conditions (see §4). In the second, we amputated gills on the left side of segments A2−5, at their base, at 21 days post hatching (dph). The non-amputated contralateral gills served as internal controls in the operated individuals (figure 1*a−c*). We then followed the growth of individual nymphs and collected the shed exuviae at each moult, on which we could measure the growth trajectories for each nymph until metamorphosis (note that following metamorphosis aerial adults lose the gills). We measured the areas of both the large and the small gill lamellae in control, regenerating and contralateral gills (figure 1*d*; electronic supplementary material, dataset S1). In addition, we measured the length of the femur from the third thoracic segment (‘hindlimb’) as a proxy of organismal size in the control and operated groups (figure 1*e*; electronic supplementary material, dataset S1).

When we plotted the area of growing gills versus time, we observed that growth followed a logistic curve, with an early acceleration, an intermediate phase with an approximately constant growth rate and a deceleration towards the end of development (electronic supplementary material, figure S1). When plotting the logarithm of the area, these growth trajectories showed a linear relation with time, which allowed us to compare them easily with the growth trajectories of the regenerating and contralateral gills of operated nymphs. This comparison showed that the growth rate of regenerating gills is significantly higher than that of their contralateral controls, while the growth rate of these latter gills is indistinguishable from the rate of unoperated controls (figure 1*d*; electronic supplementary material, figure S2A,B). Despite their accelerated growth, however, at the end of the nymphal period, the area of regenerating gills remained slightly smaller than that of contralateral or unoperated controls (electronic supplementary material, figure S3A), likely because the time left for regeneration from 21 dph to metamorphosis was not sufficient for the regenerating gills to fully catch up (electronic supplementary material, figure S2A). When we compared the overall body growth of control and amputated individuals, using femur length as a proxy or non-amputated gills from segments 1, 6 and 7, we observed that their growth trajectories overlap and reach approximately the same length at metamorphosis (figure 1*e*; electronic supplementary material, figures S1B, S2C,D and S3B). Our experiment showed that regenerating gills increase their growth rate without any noticeable effect on the overall growth of the injured animal. During our experiment, control or regenerating nymphs showed no significant differences in their developmental parameters (electronic supplementary material, dataset S2 and figure S4): for both groups, the mean time to reach the last nymphal stage was 29 days and to emergence was 34.5 days (electronic supplementary material, figure S4A,B). Both groups showed a variable number of moults, but their mean number was, again, similar (14.3 and 14.8 days for controls and regenerated nymphs, respectively; electronic supplementary material, figure S4E,F). Controls and regenerated nymphs took 2.2 days to moult (2.4 days if the last nymphal period is included; electronic supplementary material, figure S4C,D). The last nymphal instar was longer than the previous moults (5.5 days; electronic supplementary material, figure S4F), but again, its mean value was the same for controls and regenerated nymphs.

### 2.2. Faster growth is due to increased rates of cell proliferation and cell expansion

**Figure 2.**
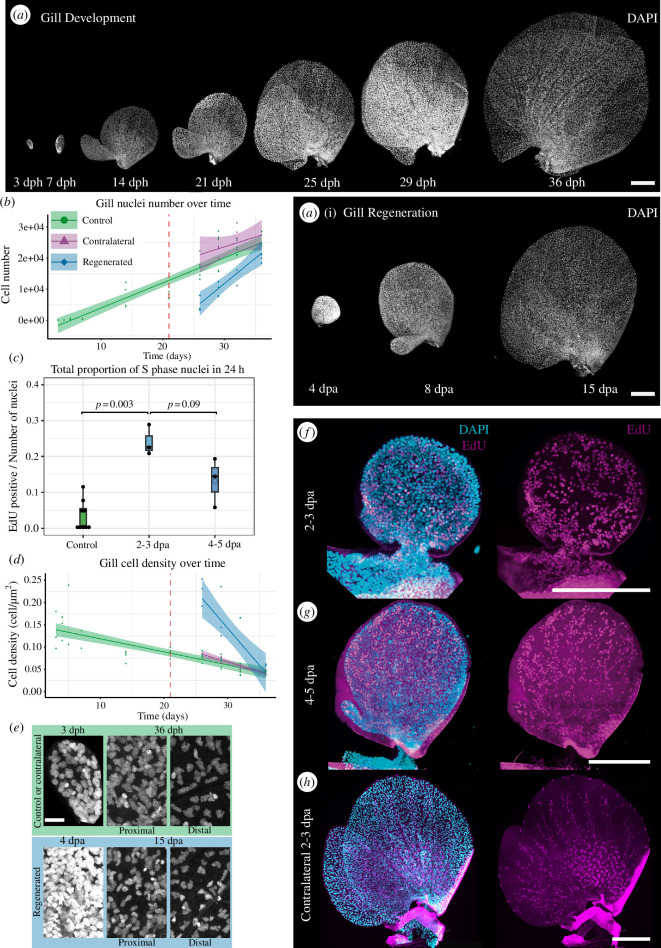
Accelerated proliferation and cell growth in regenerating gills. (*a*) Maximal z projection of DAPI (4′,6-diamidino−2-phenylindole) stained gills during development (3, 7, 14, 21, 25, 29 and 36 days post hatching (dph). (*a’*) regenerating gills at 4, 8 and 15 days post amputation (dpa) which corresponds to 25, 29 and 36 dph, respectively. (*b*) Linear models of control (green), contralateral (purple) and regenerated (blue) gills representing number of nuclei over time (*R*^2^: 0.8098, *p*‐value = 2.2 × 10^−16^. (*c*) Box plot representing the number of EdU positive cells over the total number of nuclei. Control gills: 3.35% of mean EdU positive cells; regenerating gills at 2–3 dpa: 24.08%; regenerating gills 4–5 dpa: 13.19%. (*d*) Cell density (number of nuclei divided by total area) over time (*R*^2^: 0.7205, *p*‐value = 1.086 × 10^−15^. (*e*) Close-up from panel (*a*) of the first gill in development (3 dph) and regeneration (4 dpa), and a fully developed gill of LNI contralateral and regenerated, from the proximal and distal part. The control panel is in green and the regenerating is in blue. Scale bar 10 μm. (*f–h*) Confocal images of all nuclei (DAPI staining in cyan) and the positive cells of 24 h EdU incorporation assay (magenta) for gills of different regenerated stages. (*f*) First moult after amputation (2–3 dpa). (*g*) Second moult after amputation (4–5 dpa). (*h*) Contralateral gil at (2–3 dpa). Scale bars: 100 μm.

The growth of the gill area can result from cell proliferation, cell area increase or both. In order to determine whether an increased cell proliferation rate contributed to the faster growth of regenerating gills, we repeated the experiment described above, but sacrificed the nymphs at specific time points (moults) and counted the total number of cells in each gill using DAPI staining (figure 2*a-a′*; electronic supplementary material, figure S5B; see §4). In this experiment, we corroborated the results presented earlier (electronic supplementary material, figure S5A) and observed that the rate at which cell number increased is higher in regenerating gills than in controls (either contralateral gills or those from non-operated nymphs) (figure 2*c*; electronic supplementary material, dataset S3). In order to determine whether growth resulted from localized proliferation in a ‘growth zone’ or rather proliferation was dispersed within the regenerating gill, we pulse-labelled S-phase cells using EdU injection (see §4). At 2–3 and 4–5 days postamputation (dpa) gills showed a high and uniform density of EdU-positive cells (figure 2*c*, *f*,*g*) consistent with a dispersed proliferation with a proportion of EdU-positive nuclei to total nuclei of 10–20%. By 6–7 dpa, the regenerated gills had reached almost contralateral size and trachea, chloride cells and margin bristles had already differentiated. These gills showed a much lower density of EdU cells, which were located mostly along the trachea of the large lamella and in the epithelium of the small lamella (figure 2*h*; electronic supplementary material, figure S5C and dataset S3), a result that is in agreement with the slower pace of gill growth towards the end of the regeneration process (figure 2*c*, control).

We also observed that during gill growth cell density decreased, suggesting that an increase in cell area also contributed to the overall growth of the gills (figure 2*d*,*e*). The decrease in density is observed mostly in the distal part of gills as they become flatter (figure 2*e*; electronic supplementary material, figure S5B). The fact that the decrease of cell density correlated with gill thinning (electronic supplementary material, figure S5B) suggested to us that cells flatten. Early regenerating gills had a higher cell density than control gills, even at the earliest stages, but by the end of regeneration, the average cell density was similar in regenerated and control gills (figure 2*d*,*e*; electronic supplementary material, figure S5B). From these results we concluded that, during regeneration, both the rates of cell proliferation and cell expansion are higher in regenerating than in control gills.

### 2.3. Transcriptome profiling of regenerating gills revealed changes associated with metabolism, cell proliferation and signalling pathways

To identify genes and pathways potentially involved in the regeneration process, we profiled the transcriptomes of regenerating gills (amputated at 21 dph) immediately after the first moult post-amputation at 2–3 dpa (‘early’ regeneration; figure 1*b*) and at 4–5 dpa (‘late’ regeneration; figure 1*b*). Then, we compared the transcriptional profiles of these regenerating gills with the profiles of their contralaterals (early and late contralateral groups, respectively; see §4 for details).

**Figure 3.**
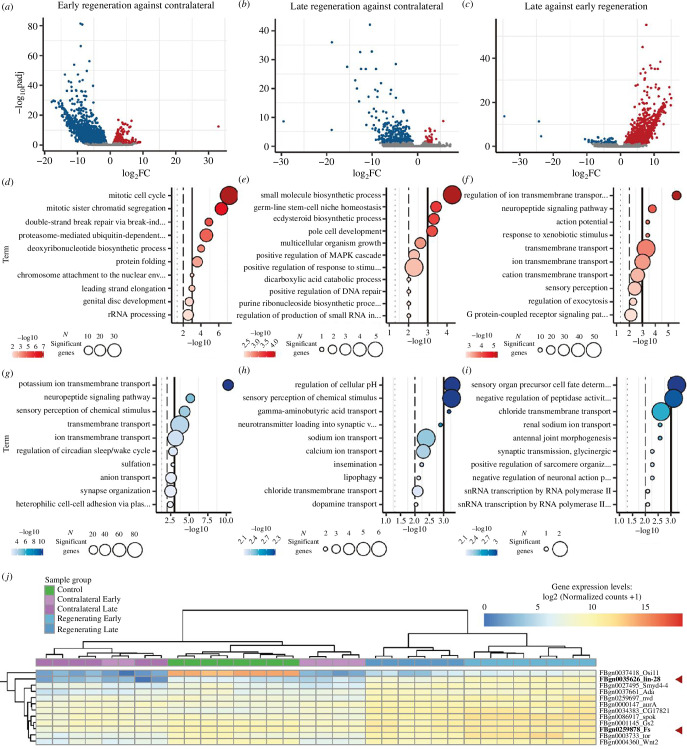
Whole gill regeneration transcriptomics. Volcano plots for different group comparisons. Blue dots represent significantly downregulated genes, and red ones significantly upregulated genes. (*a*) Early regenerating gills after first moult after amputation, against its contralateral gills. (*b*) Late regenerating gills against its contralateral gills. (*c*) Late versus early regenerating gills. Dot plots representing 10 selected enriched GO terms from upregulated genes in red and for downregulated genes in blue. (*d–f*) Significantly upregulated terms and (*g–i*) significantly downregulated terms for each of the three comparisons. The size of the dots represents numbers of significant genes in the gene ontology term, and colour indicates the *p-value* of the associated GO term in −log10 scale. (*j*) The heatmap of a small cluster of upregulated genes common to stage 1 and stage 2 that have an orthologue in *D. melanogaster* (*Osi11, lin−28, Symd4−4, Ada, nvd, aurA, CG17621, spok, Gs2, Fs, tor, Wnt2*). The colour scale represents the ‘median of ratios normalized’ (MoR) log2 transformed data, with lower expression represented in blue and higher in red. The genes marked with the red arrow (*lin-28* and *Fs*) were selected for functional testing in the *Drosophila* wing regeneration assay.

At 2–3 dpa, which represented the earliest time at which a gill rudiment is visible after amputation, we found 2232 genes that were differentially expressed in early regenerating gills relative to early contralaterals. From this set of regulated genes, 338 were upregulated and 1844 were downregulated (electronic supplementary material, dataset S5). To obtain a global view of the processes that were affected, we carried out a gene ontology (GO) enrichment analysis, using the functional annotations of *Drosophila* orthologues [28] (see Material and methods; figure 3*a,d,g*). The GO terms associated with downregulated genes (electronic supplementary material, dataset S5) were characteristic of differentiated cell types, such as transmembrane ion transport for osmoregulatory chloride cells, or synaptic organization/neuropeptide signalling for sensory cells, indicating that at this stage regenerating gill cells were less differentiated than those of the contralateral gills. The complementary set of upregulated genes (electronic supplementary material, dataset S5), even though fewer, included genes associated with mitosis and chromosomal dynamics (e.g. mitotic cell cycle/sister chromatid separation (*PCNA, Mcm, RfC3* and *RfC4*, cyclins and cyclin-dependent kinases)), chromosome attachment to nuclear envelope (*Nup62*), and DNA repair (*Ogg1*, *Rpr1* and *spn-A*)). Also, various histone genes coding for four out of five histone classes (His1, 3 genes for His2, His2A/B/H3, 3 genes for His3 and 3 genes for His4) exhibited upregulation (with none displaying downregulation), and *Nap1*, a core histone chaperone involved in histone nuclear transfer and chromatin assembly [38], was also upregulated. These transcriptional changes are indicative of increased chromatin synthesis and fast mitosis, and are in agreement with the fast proliferation of regenerating cells we observed (figure 2*c*,*f*–*h*). During this early stage of regeneration, we also observed a notable upregulation of histone methyltransferase and deacetylase genes, while genes related to histone acetylation were downregulated. This pattern suggested the prevalence of histone methylation marks during this early proliferative stage. The upregulation of *Su(Var)3-9, Syd4-4* and *PR-Set7* could induce the methylation of H3K27, H3K9 and H4K20, respectively [39–42] (electronic supplementary material, dataset S5). The transcriptional modulation of these epigenetic regulators is likely linked to the major gene expression changes detected.

In addition, two other sets of GO terms were enriched, one related to ribosomal RNA biosynthesis, presumably to sustain faster cellular growth, and the other to proteostasis (‘protein folding’ and ‘proteasome’). The latter included genes encoding components of the ubiquitination and neddylation protein-modification machinery, which were either upregulated (e.g. *SCCRO, Cand1, TER94* y *Gint3* for ‘cullin regulation and neddylation’ or *APP-BP1* and *UBA3* for ‘E1 neddylation enzymes’) or downregulated (e.g. *Roc1a* as ‘part of SKP cullin ring’, and *LUBEL* and *KLHL18* for ‘E3s of cul3’; electronic supplementary material, dataset S5).

When we looked for changes in components of major signalling pathways, we observed that elements of the Wnt (*wg, Wnt2, Wls, Gint3*), Hedgehog (*cubitus interruptus*), TGF-β (*Tgf-β like*) and insulin (*Alk*) pathways are upregulated, while components of the Dpp/BMP (*dpp* and *dally*) and FGF (*breathless*) pathways are downregulated (electronic supplementary material, dataset S5). The downregulation of the FGF receptor (*breathless*) and of the transcription factor *trachealess* likely reflect the absence of trachea at this early stage (both are associated with tracheal development in *Drosophila* [43]; electronic supplementary material, dataset S5).

Globally, the transcriptome of early regenerating gills was characterized by an upregulation of genes involved in cell proliferation, changes associated with major signalling pathways and the activation of the proteostasis machinery.

At late regenerating (4–5 dpa), when differentiated structures such as margin sensillae, tracheae and chloride cells started to become visible in regenerating gills (figure 1*b*; electronic supplementary material, figure S6A–S6L), the transcriptional profile of late regenerating gills showed fewer differences from late controls: 365 genes were differentially expressed, including 32 upregulated and 333 downregulated genes (figure 3*b,e,h*; electronic supplementary material, dataset S6). Accordingly, although late regenerating gills were still proliferating faster than controls, they did so slower than early regenerating gills (figure 2*b*, *c*,*f*–*h*). As a result, GO terms related to cell proliferation were no longer significantly enriched. The signalling pathways that we found to be up- or downregulated at early regenerating gills were also no longer differentially expressed relative to controls at late regenerating gills (electronic supplementary material, dataset S6).

When the transcriptomic profiles of late versus early regenerating gills were compared (figure 3*c,f,i*), we observed a clear transition towards a more differentiated state, e.g. GO terms for neuropeptide signalling, sensory perception or ion membrane transport were enriched in the later stage (figure 3*c*,*f*; electronic supplementary material, dataset S7). These transcriptional differences included the upregulation of several genes related to neural functions, including several Ig-containing proteins found in neuronal synapses, neuropeptides and neuropeptide receptors (electronic supplementary material, dataset S7). Ion transporters, likely linked to the differentiation of neurons and/or chloride cells, and others in *Drosophila* are required for trachea formation and branching, were also upregulated relative to earlier stages (electronic supplementary material, dataset S7). Genes associated with these GO terms, however, were still expressed at lower levels in late regenerating gills relative to their controls (electronic supplementary material, dataset S6), suggesting that these regenerating gills were not yet fully differentiated. Therefore, the transition from early to late regenerative stages is marked by the initiation of cell differentiation and a loss of the transcriptional signature of cell proliferation.

Finally, we also identified a set of genes that were consistently up- or downregulated in both early to late regenerative gills (figure 3*j*; electronic supplementary material, figure S7). Among these, 27 genes were upregulated relative to controls (electronic supplementary material, dataset S8). These might represent a core set of genes required for the initiation and maintenance of regeneration in *Cloeon* gills. They included genes with identifiable UniProt domains and *Drosophila* homologues, as well as *Cloeon*-specific genes for which no functional annotation is available. Among those with *Drosophila* homologues, this set included genes involved in metabolism (*Gs2* (glutamate synthase), *nvn* (cholesterol 7-desaturase), *spok* (cytochrome P450), *CG17821* (very-long-chain 3-oxoacyl-CoA synthase), signalling (*tor*, *Wnt2*, *Fs*), mitosis (*AurA* (aurora kinase)) and gene transcription regulation (the H3K4 methyltransferase and transcriptional repressor *Smyd4-4*) (electronic supplementary material, dataset S8).

### 2.4. Genes associated with gill regeneration in *Cloeon* identify homologues required for wing disc regeneration in *Drosophila*

In order to explore whether the function of the genes we identified in our transcriptomic analysis is conserved in insects, we knocked down a set of candidate genes during *Drosophila* imaginal disc regeneration (electronic supplementary material, dataset S9, which includes a list of references for the RNAi lines we use here; [44–46] and §4). Candidate genes were selected based on their upregulation in one or both regeneration stages. From these, we prioritized those that had been previously identified as differentially expressed during wing disc regeneration in *Drosophila* as per [16]. The list was also curated considering the existing literature, aiming to broaden the range of potential functions involved in regeneration. Our list included: *lin28*, encoding a small RNA-binding protein involved in stem cell maintenance in *Drosophila* [47–49]*,* which potentiates insulin-like signalling in the intestinal stem cells by binding the InR mRNA; the activin/TGFβ pathway component *Follistatin (Fs)*, encoding an activin repressor [50,51]; the proteostasis-related *Hsp83* and *Hsp110* chaperones; *Nedd8* and *csn5*, encoding the ubiquitin-like protein Nedd8 and a deneddylase that deconjugates Nedd8 from cullins, respectively [52,53]; the heparin-binding extracellular ligands Miple1 and Miple2 [54] and the transcription factors *E2F1* [55] and *tfb4* (part of the RNApol_II holoenzyme [56]).

**Figure 4.**
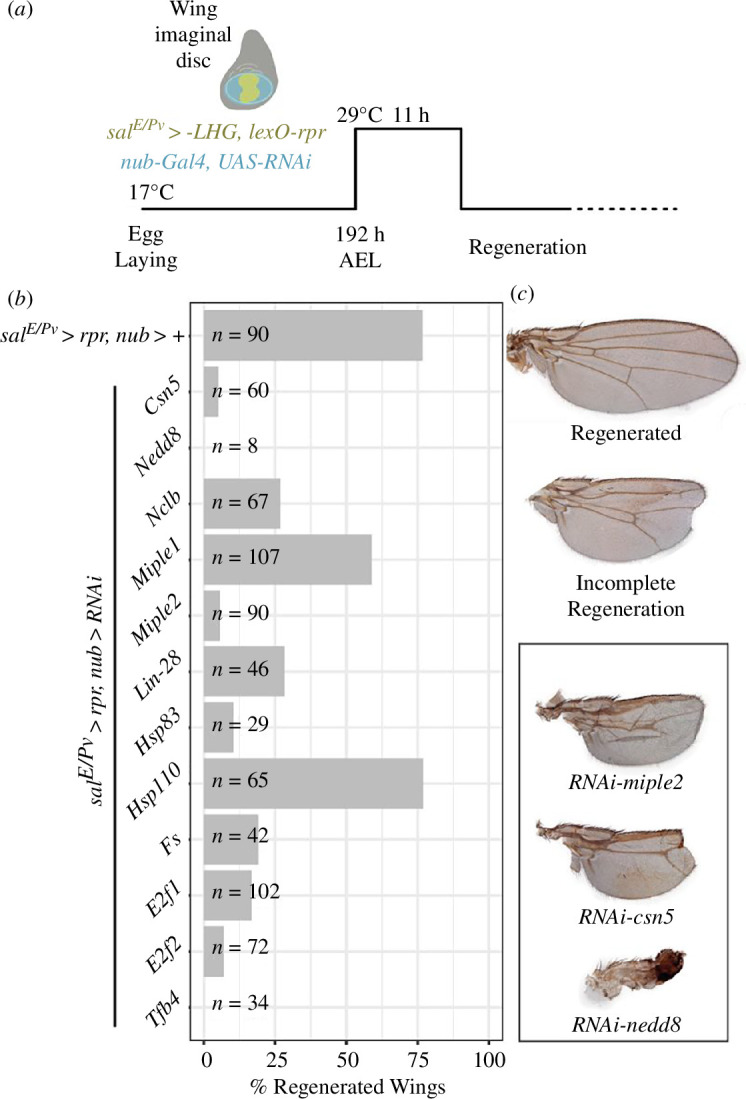
Dual transactivation experiment in *Drosophila melanogaster* wing disc regeneration. (***a***) Schematic representation of regeneration assays in wing disc regeneration using lexA/lexO and UAS/Gal4 systems. Reaper (rpr) expression is induced in the spalt wing disc domain ((a) yellow region) after a temperature induction (29°C), which degrades for that time a temperature-sensitive version of Gal80 protein (Gal80TS), which is expressed under the ubiquitous tubulin promoter. When this is performed on *Drosophila* larvae, 196 h after egg laying (AEL) for 11 h, cells within the spalt domain undergo apoptosis but surviving remaining cells proliferate to compensate lost cells and wings develop normally 77% of the cases ((*b*) nub>+, (*c*) regenerated wing). However, when also expressing a transgene under the upstream activating sequence (UAS) by using Gal4 yeast transcription factor expressed under the nubbin promoter in the wing disc pouch ((*a*) blue domain in wing disc), the wing disc may suffer defects in regeneration ((*c*) incomplete regeneration). (*b*) Histogram of percentage of fully regenerated wings with induced apoptosis and expression of RNAi for selected genes. *nub>+* indicates negative control with no RNAi induction that regenerates 80% of the cases. The number of wings examined (*n*) is indicated on each bar. (*c*) Microscope images of fully regenerated wings, incompletely regenerated wings and three cases with the most extreme defects in wing regeneration, induced by RNAis against *miple2*, *csn5* and *Nedd8*.

In this assay, a transient pulse of the pro-apoptotic gene *reaper (rpr)* damages the wing primordium in a band of cells. Following this damage, the tissue regenerates during the last part of the larval period plus the pupal stage, so that adults develop wings of size and morphology similar to control flies. The function of our selected candidate genes was tested by silencing their expression during the apoptosis-induced period and checking defects in the regeneration of the wing [44] (figure 4*a*; electronic supplementary material, dataset S10). Of this list, neither *Nedd8* nor *csn5* had been detected as upregulated. However, since neddylation pathway genes were enriched in regenerating gills, we decided to target the pathway by knocking down *Nedd8,* which codes for Nedd8 itself, and the neddylation pathway regulator *csn5.* The RNAi knock-downs against eleven of the twelve genes interfered with the regeneration in the apoptosis-induced regeneration assay (figure 4*b*), while having subtle or no effect when applied in discs without *rpr* expression (electronic supplementary material, figure S8A’–S8L’). Control wing discs, in which the pulse of apoptosis was followed by regeneration without RNAi silencing of any gene, produced morphologically normal wings in 77% of the flies, while the remaining 23% of wings showed vein fusions, or blade notching (figure 4*b*,*c*, *n* = 90; electronic supplementary material, figure S8M,N). In any case, we never observed a strong reduction of the size of the wing blade (figure 4*c*). In contrast, the defects caused when candidate genes were silenced were specific for each of them, highly penetrant and significantly stronger than controls (figure 4*b*,*c*). In particular, knocking-down *tfb4, e2f1, e2f2, miple2, csn5* and *Nedd8* exhibited very strong phenotypes in all wings analysed (*n* = 34, *n* = 102, *n* = 72, *n* = 90, *n* = 60 and *n* = 8 respectively). Interestingly, knocking down *miple1* (*n* = 107) did not produce as strong and consistent disruption in regeneration as *miple2. Hsp110*, contrary to the rest of the tested genes, had completely regenerated wings in a similar proportion to the control (77% in both). These results indicated that differential gene expression during *Cloeon* gill regeneration is a good predictor of genes required for wing disc regeneration in *Drosophila* and importantly, they pointed to a conserved role of some of these factors in appendage regeneration in insects.

### 2.5. Blocking neddylation impairs growth in regenerating gills

**Figure 5.**
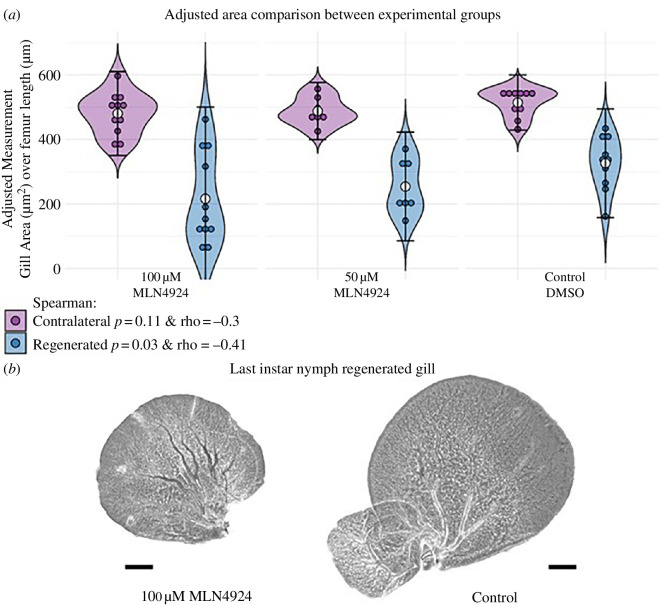
Neddylation impairment in gill growth regeneration. (*a*) Violin plot representing adjusted gill size (gill area/femur length) for amputated nymphs that were injected 48 h post amputation with three different treatments (100 μm or 50 μm of MLN4924, or DMSO as control). Contralateral gills are represented in purple and regenerated gills in blue for each treatment and condition. A Spearman rank correlation test was used showing the significance and an inverse correlation between the size and concentration of the drug used (*p*‐value = 0.03009 and ρ = −0.41). (*b*) Last instar gills: regenerated of the 100 μm group (left) and of the control group (right). Images are representative of gills with an area close to the mean area of each group. Notice that the small lamella of 100 μm is above the big lamella. The scale bar represents 100 μm (100 μm: *n* = 11; 50 μm: *n* = 7; control: *n* = 10).

The neddylation pathway has been found to be involved in many processes, including the regulation of metabolism, immune system function and tumorigenesis in humans (see [57] for a recent review). However, to our knowledge, its role in regeneration has not been previously reported. To further test whether neddylation was indeed required for gill regeneration, we injected the small molecule pevonedistat (MLN4924) [58,59], a well-established inhibitor of the neddylation pathway, in nymphs two days after gill amputation (carried out at 21 dph; see §4 for details). We found a significant concentration-dependent inhibition of regenerative growth when the area of regenerated gills of the last instar nymphs was measured relative to their contralateral controls (figure 5), which indicated a key role of this pathway in gill regeneration.

## 3. Discussion

### 3.1. Fast growth characterizes the early regenerative phase of *Cloeon* gills

After amputation, *Cloeon* gills regenerate through an early acceleration of growth, which is largely explained by an increased proliferation rate, accompanied by progressive thinning of the epithelium and lower nuclear density, which is indicative of an increase in cell area. The fast proliferation seen in the early regeneration stages correlates with the elevated expression of cell cycle-related genes, epigenetic modifying enzymes and components of growth-promoting signalling pathways (figure 3; electronic supplementary material, dataset S5). The latter include the insulin, Wnt and activin pathways, and the RNA pol I regulator *nclb,* which lies downstream of the Myc and TORC cell growth pathways [60–62] (electronic supplementary material, figure S9).

Our study does not reveal a systemic effect on the growth of the nymphs with clipped gills. Specifically, there is no developmental delay or change in the growth of uninjured gills or hindlimbs in the operated animals (figure 1; electronic supplementary material, figures S2,S3). However, our experiments were not designed to investigate the potential systemic effects of gill regeneration and unseen effects on other organs may exist [63]. Indeed, we detected a series of deregulated genes whose products could be involved in inter-organ communication due to the endocrine and paracrine functions described for their homologues in *Drosophila*. First, we detected the upregulation of *neverland* (*nvl*) and *spok*, encoding two enzymes belonging to the Halloween gene family, known for their role in the early synthesis of ecdysone (reviewed in [64]), while the ecdysone receptor (*EcR*) and its co-receptor taisman (*Tai*) were downregulated (electronic supplementary material, datasets S5, S6). Second, we identified a group of downregulated neuropeptide and neuropeptide receptors-encoding genes (neuropeptide signalling pathway_GO:0007218, like *AstC-R2*, *CapaR*, *CCHa1*, *CCHa1-R*, and *Dh31* among others; electronic supplementary material, datasets S5–S7). These gene expression changes might be indicative of endocrine or paracrine functions of regenerating gills.

### 3.2. Proteostasis and neddylation are implicated in gill/wing regeneration

During gill regeneration, many genes coding for components of the proteostasis machinery become upregulated (figure 3; electronic supplementary material, dataset S5). Indeed, mounting evidence indicates that the ubiquitin-proteasome system has an important role in stemness maintenance (reviewed in [65]), and some work has found evidence linking this system to regeneration (e.g. planaria whole body [66], sea cucumber intestine [67] and axolotl limb or zebrafish axons [68]). Our work identifies an additional mechanism of proteostasis control, neddylation, a process by which the ubiquitin-like Nedd8 peptide is covalently attached to target protein substrates [52]. The major neddylation targets are cullins, but neddylation affects other proteins as well, such as p53 or ribosomal proteins [69,70]. The list of genes implicated in neddylation which are upregulated in regenerating gills (see electronic supplementary material, dataset S5) includes *UBA3, Cand1, SCCRO*; also *Gint3 and TER94* which have been involved in Wnt signalling activation via protecting the nuclear transducer Arm from degradation by the proteasome [71]. The deregulation of the neddylation pathway during wing disc regeneration in *Drosophila* (by knocking down *Nedd8* or *csn5,* an isopeptidase that de-neddylates cullins [72]) causes a dramatic impairment of wing disc regeneration (figure 4).

This functional involvement was confirmed during gill regeneration in *Cloeon,* where pharmacological inhibition of the neddylation pathway impaired the regenerative growth of gills (figure 5). What role could proteostasisplay during regeneration? In regenerating gills cells proliferate fast, accelerating their cell cycle. This cell cycle acceleration demands fast turnover of many proteins, including cyclins and CDKs that are known substrates of the ubiquitin/proteasome system [73]. In addition, regeneration requires cells to undergo swift state transitions, including their entry into a temporary multipotent state [74]. These transitions need some degree of epigenetic reprogramming. This likely demands the regulation of chromatin-modifying enzymes, something we detected in our transcriptomic analysis (figure 3; electronic supplementary material, dataset S5). But beyond chromatin states, reprogramming must require the clearance of old proteins and the proper folding and stabilization of newly synthesized ones. Only through this proteasome dynamics, which would involve the action of chaperones, ubiquitination and neddylation, regeneration could take place efficiently.

### 3.3. Re-use of developmental programmes or regeneration-specific programmes?

One relevant question for understanding the mechanisms of regeneration is whether organ regeneration redeploys programmes that operate during development or instead calls genes and pathways that are regeneration-specific. As mentioned above, ubiquitination and neddylation are known to be very pleiotropic. However, the *Drosophila* wing disc regeneration assays showed that while the transient attenuation of genes such as *Nedd8, csn5* or *Hsp83* does not have any noticeable effect on the development of uninjured wing primordia at larval stages (electronic supplementary material, figure S8), this transient attenuation dramatically impairs wing regeneration (figure 4). Therefore, it seems that the regenerative process is more sensitive to the alterations of this pleiotropic pathway than normal organ development. This may be the case for many of the genes and pathways we find regulated during regeneration. However, we also noted that reported *Drosophila* null mutants for *lin28, Fs, miple1* or *miple2*—which are upregulated during *Cloeon* gill regeneration—are morphologically normal [47,48,54,75], indicating that these genes play minor roles, if any, during wing development. This fact suggests that some regeneration-associated genes are indeed required specifically for wing regeneration. Our functional studies have not been comprehensive, but we believe that cases such as the ones we report here support the notion that the regenerative programme of an organ utilizes genes that are not normally involved in its development. This, in turn, would imply a significant overhaul of the gene regulatory networks controlling organ development for regenerative purposes.

Finally, our functional results using *Drosophila* wing discs indicate a substantial degree of conservation for genes and processes involved in appendage regeneration of insects, since flies and mayflies diverged about 400 Myr.

### 3.4. *Cloeon* as regeneration model

This study is the first to investigate the genetic basis of *Cloeon* remarkable regeneration ability. This has been possible thanks to the relative ease of its culturing in the laboratory and the existence of a well-annotated genome that is relatively compact for an insect [28]. Techniques such as antibody staining and *in situ* hybridization have been established [20], although they are most effective in tissues not covered by cuticle (e.g. brain). Still, to make *Cloeon* a genetic model, tools such as RNAi or CRISPR need to be developed. At least for RNAi, *Cloeon*’s genome harbours homologues of genes required for RNAi processing (*Dicer, Argonaute* (*AGO1* and *AGO2*), *R2D2, Tudor-SN, Fmr1*), making the development of this technology feasible. The fact that *Cloeon* is ovoviviparous (fertilized eggs are retained within the female’s abdomen, to be laid just before the swimming nymphs eclodes) will require developing methods for maternal delivery, or adapting techniques from other insects, such as direct parental CRISPR (DIPA-CRISPR) [76] or receptor-mediated ovary transduction of cargo (ReMOT) [77]. We expect that the regeneration capacities of *Cloeon,* its phylogenetic position within insects and the possibility of developing genetic tools will stimulate its further development as a model.

## 4. Materials and methods

### 4.1. *Cloeon* culture and amputation procedure

*Cloeon dipterum* culture was as previously described [20]. All experiments were carried out in a room at 21 ± 1°C. Cold treatment (placement of dish on ice) was used for anaesthetizing the nymphs for either gill amputation, dissection or nymph injection. Gills were amputated by pulling the gill with forceps at its base. Amputation was carried out in nymphs 21 dph in which the wing pad had grown over the first half of the first abdominal segment. Gills 2–5 from the left flank were amputated except in RNAseq experiments, in which the sixth gill was also included.

### 4.2. Growth dynamics: exuviae fixation, imaging, quantitation and analysis

To follow the growth dynamics of regenerating and non-operated gills, newly hatched nymphs were separated individually in 24 well plates. Wells were checked daily for shed exuviae, which were collected for fixation so that, by the last moult, all dated shed cuticles for each individual nymph had been collected. During the experiment, the water was aired by bubbling with a Pasteur pipette. Every other day one-third of the water was replaced. Algae were kindly provided by the Aquatic Vertebrates Platform facility at the CABD and used as food which was added daily. After about two weeks after hatching, nymphs were transferred to 50 ml beakers. Both the 24 well plates and the beakers were placed in a tray with water to make the temperature even for all individuals. Exuviae were fixed in 4% formaldehyde for 20 min at room temperature or overnight at 4°C (without shaking). Then they were rinsed 3× in PBS and mounted in 80% glycerol in PBS. Images were taken in a Leica DM500B microscope with a Leica DFC490 digital camera. Measurements of the gill area were done manually using the polygon selection ROI tool from FIJI [78]. Data analysis and plots were made in R and RStudio.

### 4.3. DAPI staining, image collection and image data analysis (TrackMate and R)

For nuclei counting, nymphs were taken from different time points (1, 2, 4, 5, 7, 14, 21, 23, 26, 29, 32 dph and last nymphal instar (LNI)). The gut was removed and then nymphs were fixed in 4% formaldehyde in PBS overnight at 4°C with shaking. They were then rinsed 4 × in PBT and finally stained with DAPI (1 : 10.000) and phalloidin-488 overnight at 4°C with shaking. After the DAPI staining, samples were rinsed again 3× in PBT and 2× in PBS and equilibrated in 80% glycerol in PBS. The A3 gill was mounted and imaged in a Leica Stellaris confocal set-up, using a 63× immersion objective, as z-stacks at the Advanced Light Microscopy and Image Analysis ‘ALMIA’ platform, at the CABD (Centro Andaluz de Biología del Desarrollo). Nuclei in the image stacks were counted using the TrackMate plugin [79] for FIJI. Data were analysed with R and RStudio.

EdU injections were done using a microinjector (Narishige IM-300) and a stereomicroscope (Leica KL300) and with the help of forceps to open the needle end to an adequate size opening (1.0 mm outer diameter, 0.58 mm inner diameter, 100 mm length; 30-0016 Capillaries GC100-10 Harvard Apparatus). Needles need to be prepared from glass capillaries with the flaming/brown micropipette puller (or similar horizontal pullers) and conditions may change between different micropipette pullers and should be adjusted to the specific model.

Nymphs were injected with approximately 0.2–1 μl of EdU 10 μm dissolved in injection solution as in [80]. Nymphs were placed carefully lying on one side and all the water was removed from the Petri dish with a piece of tissue so that the nymphs stayed put in one place for injection. Injections were performed dorsally through the space between the T3 and T4 terguites, puncturing from posterior to anterior and maintaining the needle as parallel to the dorsal cuticle of the nymph as possible. Injected nymphs were placed back in beakers, allowing them to incorporate EdU for 24 h and then were sacrificed and fixed.

The Click-iT reaction for EdU was done with TermoFisher kit Click-iT EdU Cell Proliferation Kit for Imaging, Alexa Fluor 594 dye (cat. no. C10337). Most tissues of the nymphs stained well with the one Click-iT reaction. However, for the gills to be stained, five consecutive 1 h incubations in fresh reaction solution were needed. The Click-iT reaction was stopped by washing samples three times in PBS. Sample nuclei were counterstained with either DAPI or Hoescht. Imaging was carried out in a Leica Stellaris confocal set-up with 63 × oil-immersion objective at ALMIA, CABD.

### 4.4. RNAseq experimental design, RNA extraction, library preparation, sequencing and data analysis

For this experiment, nymphs were reared as described previously. On day 21 post-eclosion, the second to the sixth left gills were amputated, and individual nymphs were placed in separate plastic Petri dishes with algae ad libitum. Amputated gills at this stage were collected and this group of gills was called ‘control’ or ‘con0’ (electronic supplementary material, figure S10, green), this group was used as an external control and a proxy of a normal state of a gill within the whole inter-moulting period, and this group was subsequently used in normalization later on as the within-group variability was low and comparable between all groups (electronic supplementary material, figure S10E,S10F). We made observations each hour for 12 h during the whole regeneration process.

For the early regeneration stage, we took nymphs that had moulted once and had small regenerating gills (between 2 dpa and 3 dpa). Regenerating gills at this stage were called ‘early regenerating’ or ‘reg1’ (electronic supplementary material, figure S10, light blue) and their contralateral gills were used as internal controls and named early contralateral’ or ‘con1’ (electronic supplementary material, figure S10, light purple).

For the late regeneration stage we waited for the second moult, and only if in the first moult a small gill had appeared. Those nymphs that had not shown a regenerating gill after the first moult, were discarded. In this case, the regeneration group was called ‘late regenerating’ or ‘reg2’ (electronic supplementary material, figure S10, dark blue) and their contralateral gills ‘late contralateral’ or ‘con2’ (electronic supplementary material, figure S10, dark purple).

For each of the replicates, we used a total of 40 individuals (around 200 gills) for each replicate. Nymphs were placed on clean water before gill amputation. Amputated gills were rinsed in a drop of cold PBS before being introduced in a prechilled Eppendorf placed on liquid nitrogen, so a fast freeze was used to preserve the RNA and help disrupt the tissue for later RNA extraction. RNA was extracted using TRIzol and chloroform extraction. Biological replicates were sequenced twice and two specific samples three times, so a minimum of three biological replicates were sequenced at CNAG (https://www.cnag.eu/) using NovaSeq 6000 S1 2 × 50 bp paired end reads. The quality table can be found as (electronic supplementary material, dataset S4). Raw data are associated with BioProject PRJNA1077402 with a total of 15 BioSamples and 34 SRAs objects. The quality of the read libraries was assessed using FastQC. Then reads were aligned onto the genome (GCA_902829235.1, https://www.ncbi.nlm.nih.gov/datasets/genome/GCA_902829235.1/), (GCA_902829235.1_CLODIP2_genomic.fna and GCA_902829235.1_CLODIP2_genomic.gtf files)) using STAR [81]. The quality of the mapping was assessed with MultiQC. FeatureCounts was used to retrieve a count matrix of all the libraries, which had similar differences in levels of expression within groups (electronic supplementary material, figure S10A) and differed greatly in the number of counts within and between groups (electronic supplementary material, figure S10B). Nevertheless, libraries cluster relatively well before normalization (electronic supplementary material, figure S10C,D), and after regularized normalization with DESeq2, there is really good correlation within samples (electronic supplementary material, figure S10F) and low variability within groups and good separation between groups in the PCA1 and PCA2 (electronic supplementary material, figure S10E), but for the contralateral samples (purple) that clusterized together probably due to biological similarities between those two groups. Then, the DESeq2 library in R and RStudio was used to perform differential expression analysis of the samples. Functional annotation was retrieved from available data at NCBI [21] and Uniprot (https://www.uniprot.org/). To retrieve GO annotations for *Drosophila melanogaster* orthologue genes BioMart was used. TopGO was used for GO enrichment analysis with *Drosophila melanogaster* (dmelanogaster_gene_ensembl) functional annotation. Heatmaps were generated on normalized counts using ‘median of ratios normalization’. R libraries used in this analysis: AnnotationDbi, org.Dm.eg.db, GO.db, biomaRt, DESeq2, pheatmap, Rgraphviz, tidyr, dplyr, tibble, svglite, ggplot2 and stringr.

### 4.5. *Drosophila* dual transactivation experiment

The system used is a dual Gal4/LexA transactivation system by which one of the transactivations transiently induces cell death in a stripe within the developing wing (encompassing the *sal* expression domain) by the expression of the pro-apoptotic gene *reaper (rpr).* The second transactivator system allows gene expression in a larger domain (defined by *nub* expression) that includes the death-induced stripe. Therefore, gene silencing in the cells of the wing primordium that have not been forced into apoptosis tests the effect of the gene of interest in their capacity to regenerate the dead, missing cells and the final wing. This system also evaluates the effect that the transient manipulation of the gene of interest has on wing development, thereby allowing to discriminate between the effects on development from the effects on regeneration. This system has been described previously in [29].

The specific genetic strains used were: *sal^E/Pv^-LHG* and *LexO-rpr* strains for genetic ablation. The LHG is a modified version of lexA that contains the activation domain of Gal4 separated with a hinge construct. This form is suppressible by *tubGal80^ts^* [82]. The Gal-4 line used was *nubbin-Gal4* (*nub>*), which is expressed in the entire wing pouch. As both the LHG and GAL4 are suppressible by Gal80^ts^, the expression of *rpr* and the Gal4-driven UAS can be simultaneously controlled.

For this experiment*, w; nub-Gal4, lexO-rpr; sal^E/Pv^LHG, tubGal80^ts^* males were crossed to UAS-*RNAi (gene of interest)* virgin females. Crosses were maintained at 17°C. Synchronized 6 h egg laying collections (±3 h difference) were maintained at 17°C until day 8 after egg laying when cultures were shifted to 29°C for 11 h. This shift at 29°C inactivates the Gal80*^ts^* thus releasing both the LHG and the Gal4 transcriptional activators. After this 29°C pulse, cultures were returned to 17°C until flies emerged. Emerged adults carrying both *sal>rpr* and *nub>RNAi (gene of interest)* were fixed in a mixture of glycerol : ethanol (1 : 2) for 24 h. Wings were dissected in distilled water, rinsed in 100% ethanol, mounted in 6 : 5 lactic acid : ethanol and imaged under a compound microscope. Wing patterning defects were then scored.

### 4.6. Neddylation inhibition by pevonedistat injection

To inhibit neddylation, we used pevonedistat (MLN4924), a broadly used inhibitor of NAE, the Nedd8 E1-activating enzyme [59] (MedChemExpress, cat. no.: HY-70062). Pevonedistat was dissolved in DMSO to a 5 mM stock solution. This was dissolved again in PBS for the final injections at 100 μm MLN4924 or 50 μm MLN4924, or the same volume of DMSO as the 100 μm experimental group as control. Nymphs were injected 2 days post amputation in the thoracic region and allowed to grow till the last nymphal instar, when they were sacrificed and fixed as in previous experiments to mount the gills (regenerating and contralateral) and hindlimbs. Images were taken in a Leica DM500B microscope with a Leica DFC490 digital camera. Measurements were done manually using the ROI tool from Fiji [78]. Data analysis and plots were made using R and RStudio. For the statistical analysis of the data, we used gill area normalized to the femur length, to account for body size variation. All groups of gills (50 μm, 100 μm and DMSO-injected control) follow a normal distribution except for the 100 μm regenerated gills group (Shapiro *p*‐value = 0.0488). A Mann–Whitney test was performed to compare 100 μm regenerated to control (*p* = 0.072). A *t*‐test was used to compare the rest of the groups and all *p*-values fell above 0.05. A Spearman rank correlation test was used showing the significance and an inverse correlation between the size and concentration of the drug used (*p* = 0.03009 and *ρ* = −0.41).

## Data Availability

Transcriptomic raw data is associated to BioProject PRJNA1077402 with a total of 15 BioSamples and 34 SRAs objects in NCBI Supplementary material is available online [83].
